# Double mutants of TTLL glutamylase genes have little to no difference in viability or brood size

**DOI:** 10.17912/micropub.biology.000156

**Published:** 2019-08-29

**Authors:** Pooja Shah, Monica Martinez, Monica Chakraborty, Sharwani Kota, Bhumi Shah, Jessica Lee, Nina Peel

**Affiliations:** 1 The College of New Jersey, 2000 Pennington Rd, Ewing, NJ 08618, USA

**Figure 1. Embryonic viability and brood size of double mutants.**
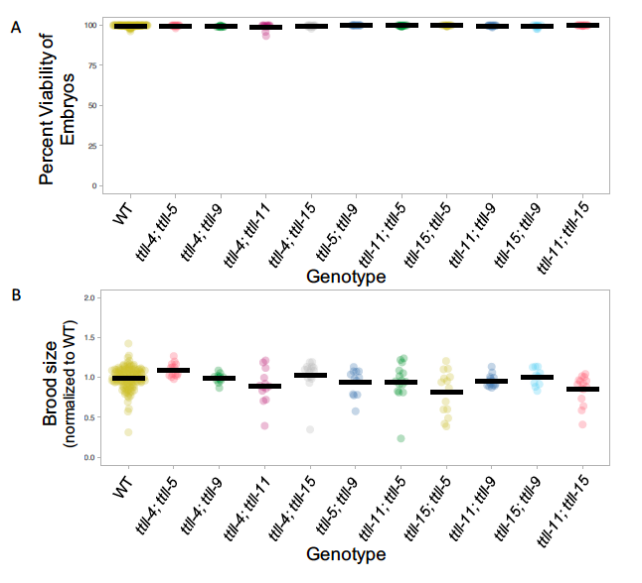
A) Embryonic viability was determined at 20°C for a minimum of 10 worms of each genotype. All data points are plotted and the means are indicated. B) Normalized brood size at 20°C is indicated for each double mutant. For each worm, brood size was normalized to the mean value of matched wild type controls. A minimum of 10 worms were assayed for each genotype. All data points are plotted and the means are indicated. Data was formatted using Plots of Data (Postma and Goedhart, 2019).

## Description

The tubulin tyrosine ligase like (TTLL) family of enzymes is required for microtubule glutamylation (Janke et al., 2005). Disruption of microtubule glutamylation has been associated with impaired centriole integrity, cilia degeneration, and aberrant neuronal function (Bobinnec et al., 1998; Gadadhar et al., 2017). *C. elegans* has five glutamylating TTLL enzymes (Chawla et al., 2016). Deletion of individual TTLL enzymes does not impair viability or brood size, moreover microtubule morphology in the early embryo is unaffected (Chawla et al., 2016). To test for potential redundancy between TTLL enzymes we constructed all combinations of double TTLL mutants. Double mutant genotypes were confirmed by PCR. Embryonic viability (percentage of embryos hatching), and brood size (the total number of embryos produced per worm) were determined over the entire reproductive lifespan for a minimum of 10 worms for each genotype, and compared with N2 worms as a wild type control. All experiments were carried out at 20°C.

The viability of the double mutants does not differ significantly from that of wild type in any case (Figure 1A, p>0.05; Student’s t-test). The average brood size for wild type worms varied between trials (ranging from 243 to 338). This presumably results from environmental fluctuations that are beyond our control such as humidity, plate batch variability, etc. To allow comparison of brood size across experiments we normalized brood size of each worm to the average brood size of controls from the same trial (Figure 1B). The brood size of the double mutants does not differ significantly from wild type with two exceptions: *ttll-5 ttll-15* (p=0.04; Student’s t-test) and *ttll-11; ttll-15* (p=0.01 Student’s t-test). Although in both cases a greater variability in brood size is observed, in neither case is there a consistent increase or decrease in fertility. In the mouse TTLL-5 and TTLL-11 preferentially modify a-tubulin (van Dijk et al., 2007). The specificity of TTLL-15 has not been tested, however it is most similar to TTLL-5 suggesting a likely preference for a-tubulin as a substrate (Chawla et al., 2016). It is therefore possible that the differences in brood size that we have observed reflect redundancy in function between TTLL-15 and other a-tubulin-modifying enzymes. Because this idea is based in an inferred function for TTLL-15, further investigation would be required to confirm the hypothesis. Although we do not find evidence of redundancy between TTLL-5 and TTLL-11, an obvious next step would be to determine whether combined loss of all three a-tubulin-modifying enzymes results in a more severe effect on fertility.

In sum, our data suggest that combined loss of any two glutamylating TTLL enzymes does not impact viability, from which we can infer that cell division, and embryonic development are unperturbed. The fertility of double mutants is largely similar to wild type, although for two genotypes a greater variability in brood size is observed. Since in neither case a consistent increase or decrease is observed the biological significance of this result is currently unclear, nevertheless it may point to a potential function for glutamylation in the germline.

## Reagents

**Table:** 

**Strain Name**	**Genotype**
NIN3	*ttll-11(tm4059) IV; ttll-15(tm3871)*
NIN7	*ttll-4(tm3310) III; ttll-15(tm3871) V*
NIN22	*ttll-5(tm3360) ttll-9(tm3889) V*
NIN26	*ttll-5(tm3360) ttll-15(tm4957) V*
NIN57	*ttll-15(tm4957) ttll-9(tm3889) V*
NIN61	*ttll-11(tm4059) IV; ttll-9(tm3889) V*
NIN62	*ttll-4 (tm3310) III; ttll-11(tm4059) IV*
NIN63	*ttll-4 (tm3310) III; ttll-9(tm3889) V*
NIN74	*ttll-4(tm3310) III; ttll-5(tm3360) V*
OC464	*ttll-11(tm4059) IV; ttll-5(tm3360) V*

Strains have not been deposited at the CGC, but are available from the authors upon request.
